# Phone-Based Text Therapy for Youth Mental Health: Rapid Review

**DOI:** 10.2196/47250

**Published:** 2023-12-14

**Authors:** Varun Karnik, Hamish Henderson, Urooj Raza Khan, James Boyd

**Affiliations:** 1 La Trobe University Melbourne Australia; 2 Griffith University Gold Coast Australia

**Keywords:** text therapy, mHealth, adolescent health, distance counseling, mental illness, mobile health intervention, adolescent, health promotion, digital mental health intervention

## Abstract

**Background:**

Mental illness has become a prevalent issue impacting adolescents worldwide. Many barriers, including stigma and poor health literacy, prevent this population group from accessing reliable mental health care services. Synchronous text–therapy counseling is an underused therapeutic approach in combating adolescent mental illness. Phone-based text therapy is uniquely placed to offer personalized counseling to adolescents through a familiar and engaging treatment modality.

**Objective:**

This rapid review aims to understand the clinical effectiveness, usability, and accessibility of phone-based text therapy for youth mental health.

**Methods:**

Cochrane CENTRAL, Embase, PubMed, and PsycINFO were used to search for suitable literature. Five groups of keywords were used: those related to (1) “therapy,” (2) “text,” (3) “phone,” (4) “youth,” and (5) “mental health.” Eligibility criteria were formed through the PICO (Population, Intervention, Control, and Outcome) framework. Studies were included if a synchronous phone-based text therapy intervention was used in an adolescent population, with an age range of 12-24 years. Only literature available in full-text, English, and a peer-reviewed journal was considered. Furthermore, a date limit of 5 years was set to reflect the recent development of digital interventions for mental health. Pertinent information from each study was tabulated, and a narrative synthesis was used to assess, describe, and organize the included studies comprehensively and concisely.

**Results:**

Of the 771 studies dual screened, 7 studies were included in this rapid review. Most of the exclusions occurred due to the use of the wrong intervention, such as asynchronous messaging. The selected studies had a low risk of bias and were suitable for the review. All interventional trials demonstrated reductions in mental health symptoms, primarily depression and anxiety. Most studies displayed high usability among participants, while data were unclear regarding accessibility.

**Conclusions:**

This review reveals the high potential of phone-based text therapy as an intervention for adolescents experiencing mental illness. We hope that this review promotes further refinement of text-based phone therapies and encourages future research on this subject matter.

## Introduction

### Rationale

The rates of mental illness have increased rapidly worldwide, with 29% of individuals experiencing mental health issues at some point in their lifetime [1]. Mental disorders significantly impact the quality of life and are predicted to cost the global economy over US $16 USD trillion between 2011 and 2030 [2]. The mental health of our youth is of particular concern, as 50% of adolescents are currently experiencing mental health problems and 70% of mental disorder diagnoses occur before the age of 25 years [3,4].

Despite the numerous services offered, a large proportion of affected youth remain unsupported. Often termed the “missing-middle,” this population is not reached by mental health services due to a variety of barriers that contribute to a lower access rate. Barriers include a shortage of resources; prolonged waiting lists; poor health literacy; and concerns related to trust, stigma, and confidentiality [5-7]. While primary care–based models, such as *Headspace* in Australia, can help in the initial stage of mental health problems, evidence shows that adolescents often disengage from these services much earlier than desirable [8,9]. Furthermore, only a minority of those who remain engaged experience positive outcomes [10]. It is evident that additional services are required to provide more substantial and sustained care for our youth.

A growing field of interest is digital mental health, which encompasses any application of digital technology to the assessment, prevention, or treatment of mental health issues [11]. In a digital world, where most communication is performed on smartphones, the use of phone-based text therapy has emerged as a promising option. This has the potential to connect youth with therapists and mentors, improve accessibility to resources, and manage youth mental health through an engaging medium. Text therapy has been investigated in the literature, with a recent scoping review from Dwyer et al [12] analyzing 70 studies and concluding that text-based services can deliver effective mental health support to the population. Interestingly, the authors asserted that the assumed limitations of text therapy may act as an advantage, with increased anonymity effectively assuaging patient concerns about confidentiality and privacy [12].

While the general use of text therapy to deliver mental health support has been explored, there is limited evidence focusing on the delivery of these interventions through a phone to the adolescent population. Consequently, we performed a rapid review to collate information regarding phone-based text therapy for youth mental health, informing the design and delivery of future digital mental health interventions.

### Objectives

This rapid review aimed to collate and summarize current evidence relating to phone-based text therapy options for youth mental health. The following research questions were asked: What is the effectiveness of phone-based synchronous text therapy options for youth mental health in terms of (1) clinical outcomes and (2) usability and accessibility?

## Methods

### Eligibility Criteria

The eligibility criteria for this review were developed using the PICO (Population, Intervention, Control, and Outcome) framework. In this study, the “population” encompassed youth experiencing mental health symptoms. Youth was defined using the Australian Institute of Welfare and Health definition as individuals aged 12-24 years [13]. All mental health symptoms were included, with particular emphasis on the most prevalent symptoms of depression and anxiety [13]. The “intervention” was any phone-based text mental health therapy intervention. This intervention had to be synchronous, provided by a human therapist or support staff with adequate training, and delivered through an app or service available on a phone. Combination interventions were included if text therapy was 1 of the main components of the treatment. Where applicable, the “control” was a lack of treatment or any comparison treatment. This included individuals on a waitlist, self-help therapy, face-to-face therapy, and telehealth services. Finally, the “outcome” was the effectiveness of treatment in terms of (1) reducing clinical outcomes, such as mental health symptoms, and (2) usability and accessibility.

According to the *Cochrane Rapid Reviews Interim Guidance*, only literature available in full text, English, and a peer-reviewed journal was considered [14]. The literature was date limited to 5 years, in order to reflect the recent development of digital interventions for mental health and to ensure the services explored in the review were contemporary and comparable. Observational and experimental studies were included. Studies were excluded if they described automated, nonsynchronous, scheduled, or unidirectional messaging. Similarly, any articles that used text therapy to manage medication compliance, reminders regarding health care, or treatment adherence were also excluded.

### Information Sources

The following electronic databases were searched: Cochrane CENTRAL, Embase, PubMed, and PsycINFO. The reference lists of each article selected for full-text review were also manually searched.

### Search Strategy

Advanced search functionality was used, with searches including keyword truncations, Medical Subject Headings terms, and subject headings. An information specialist from the Griffith University assisted with the development of the strategy. The searches were conducted in December 2022, using five groups of keywords: those related to (1) “therapy,” (2) “text,” (3) “phone,” (4) “youth,” and (5) “mental health.” Full search strategies for each database are included in Multimedia Appendix 1.

### Selection Process

Literature retrieved from searches was exported into EndNote X9 (Clarivate). These were then transferred into Covidence (Veritas Health Innovation Ltd), a digital data extraction and screening tool. Duplicates were removed, and items were screened in accordance with the eligibility criteria by screening the title or abstract (1) and then by full text (2). One reviewer screened all titles or abstracts, while the full-text screening was undertaken by 2 independent reviewers (VK and HH). Any disagreements were discussed extensively, and a third reviewer (JB) was brought in if needed. The *Cochrane Rapid Reviews Interim Guidance* from the *Cochrane Rapid Reviews Methods Group* was used [14], and the selection process was recorded via a PRISMA (Preferred Reporting Items for Systematic Reviews and Meta-Analyses) chart [15] (Multimedia Appendix 2).

### Data Collection Process

Data were extracted from the literature into an Excel (Microsoft Corp) table. The extraction was completed by 1 researcher and then reviewed by another (VK and HH). If inconsistencies arose, a third researcher (JB) was involved to achieve agreement. The information extracted included (1) information about the study, including the authors, publication date, study type, and location; (2) information about the study participants, including the sample size, age, and sex; (3) for research question 1, information about the effect on clinical outcomes, primarily depression and anxiety, or any other mental health symptoms; and (4) for research question 2, information about accessibility and usability.

### Study Risk of Bias Assessment

The appropriate tool was used to evaluate the literature included in the review:

- Randomized controlled trial: risk of bias in randomized trials tool [16]
- Uncontrolled studies: a quality assessment tool for quantitative studies by the Effective Public Health Practice Project [17]
- Cohort studies: Risk of Bias in Non-Randomized Studies of Interventions (ROBINS-1) tool [18]
- Cross-sectional studies: Appraisal Tool for Cross-Sectional Studies (AXIS) [19]
- Case control studies: Critical Appraisal Programme Skills [20]
- Qualitative studies: Joanna Briggs Institute Critical Appraisal Checklist for Qualitative Research [21]
- Ecological studies: Modification of Strengthening the Reporting of Observational Studies in Epidemiology. [22]

### Synthesis Methods

A narrative synthesis was used. First, the pertinent information from each study was tabulated. Following this, the results of each included study were assessed, described, and organized in a comprehensive and concise format [23].

## Results

### Study Selection

**Figure 1:**
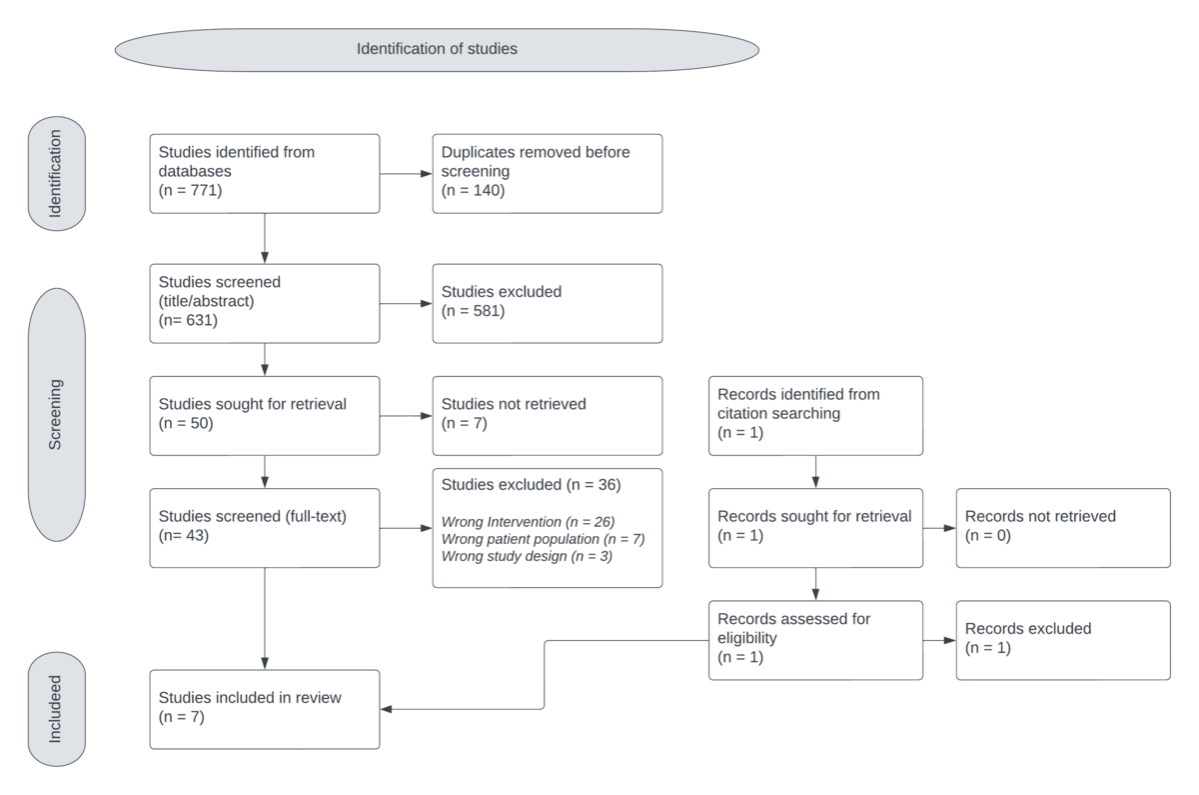
Untitled.

Studies were selected using the processes shown in the PRISMA diagram (Figure 1). Searches were executed on December 20, 2022, returning 771 citations. A total of 140 duplicate studies were eliminated, resulting in 631 studies available for initial screening. Subsequently, 581 studies were excluded based on title and abstract assessment. Additionally, 7 studies proved inaccessible for retrieval, and 31 more were eliminated after a thorough examination of their full texts. Finally, 7 studies were included in the rapid review. Studies were excluded primarily due to the implementation of the wrong intervention such as the use of automated or asynchronous messaging. Dual screening was performed for the full-text screening, with any disagreements being extensively discussed. Furthermore, the reference lists for articles selected for full-text screening were manually searched, with 1 study being assessed for eligibility, but ultimately excluded.

### Study Characteristics

Information on each study is shown in Table 1. The studies were conducted in a variety of settings: Australia [24], Europe [25,26], Asia [27], and North America [28-30]. Several trials contained both quantitative and qualitative components [24,27,28], and some were purely quantitative [25,26,29,30]. The majority (4/7, 57%) were interventional trials, with 3 uncontrolled trials [24,25,28] and 1 randomized controlled trial [26]. The remaining trials (3/7, 43%) consisted of a cross-sectional study [30], an ecological study [29], and a retrospective audit study [27].

**Table 1:** Results of interventional trials.

Reference and study characteristics	Intervention	Participants	Clinical effectiveness	Usability
Alvarez-Jimenez et al [24] (2020) Study type: uncontrolled single-group trial Setting: Australia (Oceania)	Intervention: MOST+^a^ for 9 weeks with a qualified therapist Time accessible: 4 PM and 12 AM Control: N/A^b^	N=157 Mean age 19.1 (SD 2.3) years 121 (77%) female	Statistical improvements in psychological distress, perceived stress, well-being, depression, loneliness, social support, autonomy, and self-competence after MOST+ use 82% of participants reported that intervention helped them feel better 86% of participants reported more social connectedness Correlation between self-reported benefits and web-based messages with clinicians	98% of participants reported positive experience 86% of participants reported easy to use 92% of participants would recommend it to others
Lindqvist et al [26] (2020) Study type: randomized controlled trial Setting: Sweden (Europe)	Intervention: IPDT^c^ for 8 weeks with a qualified therapist Time accessible: whenever needed Control: supportive contact over the internet	N=76 Mean age 16.6 years 80% female	Patients in the intervention group (IPDT) saw a significantly faster decline in depression symptoms compared to those in the control group improvements Intervention reduced anxiety Intervention increased emotional regulation and self-compassion Results regarding depression and anxiety symptoms were stable at follow-up after 6 months	12% of participants dropped out 3% of participants complained of loneliness when using the text-based intervention
Goldin et al [25] (2019) Study type: uncontrolled single-group trial Setting: Finland (Europe)	Intervention: ascend program for 8 weeks with a qualified therapist Time accessible: whenever needed Control: N/A	N=22 Mean age 23.2 years 100% female	Significant reduction in depression Correlation between the number of days of practice and reduction in depressive symptoms Correlation between the number of weeks of group chat use and reduction in depressive symptoms	27% of participants dropped out
Chyzzy et al [28] (2020) Study type: uncontrolled single group trial + qualitative component Setting: Canada (North America)	Intervention: MPPS^d^ for 7 months with a trained mentor Time accessible: whenever needed Control: N/A	N=21 Mean age 21.3 years 100% female	65.1% of participants confirmed improvements in stress and coping Correlation between the number of contacts and participant satisfaction 43.4% of participants confirmed that improvements in stress or coping were due to intervention 59.7% of participants confirmed that improvements in social integration were due to intervention	78.2% of participants reported convenience of support 79.2% of participants reported they enjoyed the access to support 37.5% of participants enjoyed receiving peer support by phone, particularly messaging

^a^MOST+: Moderated Online Social Therapy+.

^b^N/A: not available.

^c^IPDT: internet-based psychodynamic therapy.

^d^MPPS: mobile phone–based peer support.

### Participants

The interventional studies had a wide range of sample sizes (from n=21 [28] to n=157 [24]). The participants in the interventional trials were predominantly female, with studies by Goldin et al [25] and Chyzzy et al [28] consisting of a 100% female population, and Alvarez-Jimenez et al [24] and Lindqvist et al [26] were made up of 77% and 80% female participants, respectively. These trials all had participants of similar ages, with the mean age ranging from 16.6 [26] to 23.2 years [25]. The noninterventional trials comprised various observational studies and had larger sample sizes (n=662 [30] to n=849,483 [29]). Yip et al [27] and Thompson et al [29] lacked information about age and sex, while Toscos et al [30] slightly favored female participants, with a mean age of 20.91 years.

### Intervention Characteristics

#### Phone-Based Text Therapy

Yip et al [27] and Thompson et al [29] collated data from an adolescent population that solely used a text therapy service available on phones. These services, known as Crisis Text Line and OpenUp, are free text counseling support systems that enable adolescents to connect with social workers and volunteer counselors.

#### Combination Therapies

The other interventions largely consisted of combination digital health treatments, with synchronous text therapy comprising a significant component. Goldin et al [25] and Alvarez-Jimenez et al [24] used a combination of digital interventions named Moderated Online Social Therapy+ and the Ascend Program, respectively. These interventions consisted of 3 separate components, including a self-learning component (modules and daily exercises), a group connection (peer social network [24] and group chat [25]), and private phone–based text therapy with a qualified therapist [24,25]. Toscos et al [30] examined youth perceptions of “tele-mental health services” in their cross-sectional study, which was described as self-help materials, web-based therapy, and a phone-based text therapy service. Finally, Chyzzy et al [28] and Lindqvist et al [26] used a combination of digital interventions with 2 components named mobile phone–based peer support and internet-based psychodynamic therapy, respectively. One study used a call service and phone messaging support [28], and another used modules and phone-based text therapy [26].

#### Support Staff and Time Used

The support staff connected to participants consisting of qualified therapists [24-26] and volunteer counselors or mentors [27-29]. Several interventional trials (n=3) consisted of a use period of approximately 2 months [24-26]. Chyzzy et al [28] allowed support for 7 months, as this study focused on postpartum depression and included support before and after giving birth. The majority of the interventional trials allowed phone-based text support whenever required [25,26,28]; however, 1 study only provided access from 4 PM to 12 AM [24]. As for the noninterventional studies, Yip et al [27] and Thompson et al [29] included information from 4 and 5 years of data, respectively [27,29].

### Results of Syntheses

The results from the collated studies are outlined in Table 2. Due to the heterogeneity of the primary interventions used across the included studies, a meta-analysis was not performed.

**Table 2:** Results of included noninterventional trials.

Reference	Data studied	Participants	Accessibility and Usability
Yip et al [27] (2021) Study type: retrospective audit Setting: Hong Kong (Asia)	A survey from users of OpenUp chat services between 2018 and 2021	N=29,400 Mean age unknown Sex: unknown	From October 2018 to June 2021: OpenUp served 81,654 total sessions 13,244 (81.5%) participants found the service helpful 12,688 (85.4%) participants were likely to seek help in the future 5015 (79%) participants found the community information helpful 10,143 (34.5%) participants used OpenUp services multiple times 11,917 (45.5%) users had never sought help from others about the issue they discussed in OpenUp. 2471 (85.3%) high risk and crisis cases, in terms of suicide risk levels, were lowered by the intervention
Toscos et al [30] (2018) Study type: cross-sectional study Setting: United States (North America)	Survey of college students about telemental health	N=662 Mean age 20.91 (SD 1.69) years 438 (66%) female	88 (13.8%) participants had used text therapy before 452 (68.3%) students would prefer to talk to someone, face to face, about their stress or problems in person 111 (16.8%) students preferred text or web-based chat, 61 (9.2%) students preferred a phone call, 12 (1.8%) students preferred a video chat, 5 (0.8%) students preferred social media, and 8 (1.2%) students preferred something else Independent of depression, sex, or stress level, participants preferred face-to-face versus web-based therapy Students reported the most interest in using self-help resources (n=258, 40.1%), followed by a web-based therapist (n=184, 28.8%), followed by anonymous web-based chats with trained nonprofessionals (n=158, 24.6%)
Thompson et al [29] (2018) Study type: ecological study Setting: United States (North America)	Use of crisis text line from 2013 to 2017 in different areas	N=849,483 Mean age unknown Sex: unknown	The biggest predictor of CTL nonuse was rural communities CTL use was also associated with higher mean household incomes, higher divorce rates, and lower residential stability Did not eliminate socioeconomic disparities in service use Use not dependent upon on-ground mental health services

#### Clinical Effectiveness

All 4 interventional trials demonstrated a reduction in mental health symptoms. Improvements in depression, anxiety, psychological distress, and emotional regulation were confirmed [24-26,28]. Other reported outcomes included an improvement in social connectedness, social integration, and coping mechanisms [24,28]. Correlations between text-based messaging and mental health outcomes were reported by 2 studies [24,25]. Only 1 randomized controlled trial was included, with Lindqvist et al [26] demonstrating that patients in the intervention group had a significantly faster improvement in mental health symptoms when compared to a control group.

#### Usability

In terms of usability, most participants reported a positive experience with phone-based text therapy interventions. Users reported that text therapy interventions were easy to use (86% from Alvarez-Jimenez et al [24]), highly convenient (78.2% from Chyzzy et al [28]), reusable (34.5% from Alvarez-Jimenez et al [24]), and helpful (79% from Alvarez-Jimenez et al [24]). Notably, studies described increases in feelings of autonomy, self-competence, and self-compassion [24,26]. Conversely, in 1 study, 27% of participants dropped out [25], while in another study, 3% of participants made complaints regarding loneliness when using the intervention [26].

#### Accessibility

Regarding accessibility, most of the information provided was from noninterventional studies. Chyzzy et al [28] reported that 79.2% of users enjoyed the accessibility of the interventions. Toscos et al [30] indicated participants are more comfortable engaging in face-to-face therapy compared to web-based or phone-based therapy. When comparing all forms of digital mental health support, it was evident that 24.6% of participants preferred using text or web-based chats. Thompson et al [29] indicated that there was a higher use and accessibility of text therapy in communities with higher mean household incomes, higher divorce rates, and lower residential stability. Most notably, text-line use was not dependent upon existing physical and mental health services, and rurality was the biggest predictor of nonuse.

### Quality Assessment

All studies were subject to quality assessment with the appropriate tool, and the results are shown in Multimedia Appendix 3. All studies had a low risk of bias and were suitable to include in the review. The cross-sectional study, uncontrolled trials, and ecological study were associated with some bias due to the nature of their study types, and this was considered during interpretation.

## Discussion

### Principal Findings

This rapid review aimed to collate information regarding the clinical effectiveness, usability, and accessibility of phone-based text therapy interventions for youth mental health. This review identified 7 studies that used phone-based text therapy as a primary component in a digital mental health intervention for adolescents. Improvements in clinical outcomes were seen across all studies, particularly in symptoms of depression and anxiety. Furthermore, combination interventions using phone-based text therapy were seen to be generally usable, while evidence regarding accessibility was mixed.

Our study corroborates results from a previous review by Dwyer et al [12], which explored text-based interventions in the general population. This signifies that similar clinical benefits can be seen when applying text therapy in a younger cohort. Specifically, our review presented improvements in a wide array of mental health symptoms, including distress, well-being, depression, and anxiety. While the extensive use of combination digital health treatments limits our examination of phone-based text therapy as a sole intervention, general inferences can still be made. Notably, an included study from Alvarez-Jimenez et al [24] reported a correlation between the number of text-based messages exchanged with a clinician, and the self-reported benefit in well-being. This demonstrates that the text therapy component of the combination digital intervention was a major contributing factor to the reported clinical benefit. Goldin et al [25] presented similar results, indicating that the reduction in depressive symptoms was predicted by the use of the group chat function. While Goldin et al [25] emphasized the use of the group chat function as opposed to the private text therapy component, these results still suggest that great benefit comes from connecting patients to support via phone. In addition to improving well-being through direct counseling, phone-based text therapy enhances social connectedness to therapists, mentors, and peers [31]. This fulfills the need for belonging and security that have been shown to be extremely important in developing adolescents [31,32].

Theoretically, digital mental health interventions can alleviate stress by providing “received support,” including advice, emotional comfort, and empathy [33,34]. Interestingly, research has shown that “perceived support,” which is merely the trust that support will be readily available if needed, may have a stronger influence on mental health outcomes [33,34]. In most of the interventional trials, phone-based text therapy was available whenever required, and this “perceived support” undoubtedly had a strong therapeutic influence on the mental health of participants. The “perceived support” afforded to adolescents had multiple other benefits, with our results noting improvements in autonomy, self-competence, and self-compassion [24,26]. This suggests that the self-directedness, flexibility, and freedom of phone-based text therapy allow adolescents to be independent in the pursuit of their recovery. This could improve their self-esteem and maturity while concurrently providing the mental health therapy required.

The usability of digital interventions involving phone-based text therapy was consistently described as easy to use, helpful, reusable, and convenient. This could be attributed to the high digital literacy levels of younger people, specifically their familiarity with, and reliance on, phone-based communication [35]. A known limitation of text therapy is the difficulty in forming meaningful interpersonal relationships [36]. One study did reflect this, noting that a very small number of participants complained of loneliness despite text support being available 24/7 [26]. Evidently, while some adolescents enjoy the advantages of text therapy, others may require a therapeutic relationship characterized by face-to-face counseling. This signifies the nuances in mental health therapy and emphasizes the importance of an individualistic and bespoke approach to optimize treatment.

Our review presents varied results in terms of accessibility, with 1 study asserting that adolescents enjoyed accessing digital text interventions, while another study indicated that young college students were more comfortable engaging in face-to-face therapy. This contrasts with the literature, as multiple studies have concluded that text-based digital counseling options are more accessible to youth, due to their flexibility, anonymity, and affordability [12,37,38]. Toscos et al [30] attributed the conflicting preference for face-to-face care in their study to lack of knowledge and previous experiences. While the literature might indicate that phone-based text therapy is widely accessible for the youth population, Thompson et al [29] demonstrated that text therapy is still not well used in rural areas, even in areas with a strong established presence of physical and mental health services. This suggests that while text therapy might be more accessible to some adolescents, there are still unique barriers in rural areas that require further research [39,40].

### Limitations and Future Directions

This rapid review has numerous limitations which must be considered. First, to provide more timely information the literature search was limited in various ways: (1) the “mental health” component of the search strategy was limited to primarily depression and anxiety, (2) only 4 databases were searched, and (3) a 5-year date limit was implemented. Furthermore, there is a lack of standard nomenclature in this space and a subsequent heterogeneity of terms used to describe text-based synchronous interventions. All these factors may have contributed to some relevant studies being missed in the literature search. Additionally, the studies included were highly heterogeneous, with most using text therapy as a component of a combined intervention. This reduces the strength of conclusions made in this review, as it was difficult to elucidate the impact of text therapy as a sole treatment option. Furthermore, the interventional studies included consisted primarily of uncontrolled trials, which limits our ability to confirm clinical effectiveness. Nevertheless, the positive results in these studies do establish that there is a clinical effect worth further investigation.

Due to their cost-effectiveness and ease of implementation, we expect an increase in the use of text therapy in youth mental health. While feasibility studies show promising results, the clinical viability of this form of therapy requires further investigation. Additional research should be performed using phone-based text therapy as the sole mental health intervention in the youth population. While combination interventions have advantages, it is important to establish the effectiveness of each component to optimize these therapies. Research focusing on the perceptions of the rural youth population on phone-based text interventions must be emphasized, as these areas experience the most significant shortages in mental health resources. An improvement in our understanding of this will greatly improve future interventions.

### Conclusions

In conclusion, this rapid review provides a collation of recent research into phone-based text therapy options for youth mental health. Our review provided mixed evidence regarding accessibility but strongly indicated that phone-based text therapy improves mental health symptoms and is generally usable. We hope this review informs the design and delivery of phone-based text therapy interventions for adolescents struggling with their mental health and instigates further research in this area.

## Appendix

Multimedia Appendix 1 Search Strategies.

Multimedia Appendix 2 PRISMA Checklist.

Multimedia Appendix 3 Quality Assessments.

## Abbreviations

AXIS: Appraisal Tool for Cross-Sectional Studies
PICO: Population, Intervention, Control, and Outcome
PRISMA: Preferred Reporting Items for Systematic Reviews and Meta-Analyses
ROBINS-1: Risk of Bias in Non-Randomized Studies of Interventions
