# Supplementary material for: Phone-Based Text Therapy for Youth Mental Health: Rapid Review
Source: Interact J Med Res. 2023 Dec 14;12:e47250. doi: 10.2196/47250 (PMC10755647; doi:10.2196/47250)
Supplement: Multimedia Appendix 1 [file ijmr_v12i1e47250_app1.docx]

**Multimedia Appendix 1: Search Strategies**

PubMed search strategy:

*((((smartphone OR application OR app OR mobile* OR iphone OR "smart phone") OR (("Cell Phone"[Mesh]) OR "Mobile Applications"[Mesh])) AND ((mental OR depress* OR anxiety) OR ((("Mental Health"[Mesh]) OR "Depression"[Mesh]) OR "Depressive Disorder"[Mesh]))) AND ((youth* OR "young adult*" OR teen*) OR (("Adolescent"[Mesh]) OR "Young Adult"[Mesh]))) AND (((("Text Messaging"[Mesh]) OR (text* OR message* OR chat*)) AND (((therap* OR counsel* OR intervention*) OR ("Psychotherapy"[Mesh])) OR ("Counseling"[Mesh]))) OR (("online counsel*" OR telehealth* OR e-therapy* OR "text therap*" OR "internet-based therap*" OR teletherap* OR telecounsel*) OR (("Distance Counseling"[Mesh]) OR "Telemedicine"[Mesh])))*

APA PsycInfo search strategy

*1 (smartphone or application or app or mobile* or iphone or "smart phone").mp. [mp=title, abstract, heading word, table of contents, key concepts, original title, tests & measures, mesh word] 172180*

*2 exp mobile phones/ 7197*

*3 exp mobile applications/ 2063*

*4 1 or 2 or 3 172320*

*5 (mental or depress* or anxiety).mp. [mp=title, abstract, heading word, table of contents, key concepts, original title, tests & measures, mesh word] 1071584*

*6 exp major depression/ 152573*

*7 exp mental health/ 84326*

*8 5 or 6 or 7 1071706*

*9 (youth* or "young adult*" or teen*).mp. [mp=title, abstract, heading word, table of contents, key concepts, original title, tests & measures, mesh word] 342337*

*10 exp adolescent development/ 64390*

*11 9 or 10 381511*

*12 (text* or message* or chat*).mp. [mp=title, abstract, heading word, table of contents, key concepts, original title, tests & measures, mesh word] 171647*

*13 exp text messaging/ 1408*

*14 12 or 13 171647*

*15 (therap* or counsel* or intervention*).mp. [mp=title, abstract, heading word, table of contents, key concepts, original title, tests & measures, mesh word] 1069396*

*16 exp counseling/ 82363*

*17 exp psychotherapy/ 217885*

*18 15 or 16 or 17 1136290*

*19 ("online counsel*" or telehealth* or e-therapy* or "text therap*" or "internet-based therap*" or teletherap* or telecounsel*).mp. [mp=title, abstract, heading word, table of contents, key concepts, original title, tests & measures, mesh word] 4225*

*20 exp online therapy/ 3838*

*21 19 or 20 7477*

*22 18 or 21 1137840*

*23 4 and 8 and 11 and 14 and 18 and 22 120*

*24 23 and 4 and 8 and 11 and 14 and 22 120*

*25 4 and 8 and 11 and 14 and 22 120*

Cochrane Central search strategy

*Date Run: 20/12/2022 06:57:53*

*ID Search Hits*

*#1 (*(therap* OR counsel* OR intervention) AND (text OR messag*) AND (app OR phone) AND (youth* OR adolescents) AND (mental OR depression OR anxiety)) (Word variations have been searched) 778*

*#2 MeSH descriptor: [Cell Phone] explode all trees 2405*

*#3 MeSH descriptor: [Mobile Applications] explode all trees 1161*

*#4 #1 OR #2 OR #3 3593*

*#5 (mental OR depress* OR anxiety):ti,ab,kw (Word variations have been searched) 180385*

*#6 MeSH descriptor: [Mental Health] explode all trees 2004*

*#7 MeSH descriptor: [Depressive Disorder] explode all trees 13539*

*#8 #5 OR #6 OR #7 180439*

*#9 (youth* OR "young adult*" OR teen*):ti,ab,kw (Word variations have been searched) 105660*

*#10 MeSH descriptor: [Adolescent] explode all trees 110852*

*#11 MeSH descriptor: [Young Adult] explode all trees 72998*

*#12 #9 OR #10 OR #11 176820*

*#13 (text* OR message* OR chat*):ti,ab,kw (Word variations have been searched) 25338*

*#14 MeSH descriptor: [Text Messaging] explode all trees 1161*

*#15 #13 OR #14 25338*

*#16 (therap* OR counsel* OR intervention*):ti,ab,kw (Word variations have been searched) 1174248*

*#17 MeSH descriptor: [Psychotherapy] explode all trees 27290*

*#18 MeSH descriptor: [Counseling] explode all trees 6038*

*#19 #16 OR #17 OR 18 1263394*

*#20 ("online counsel*" OR telehealth* OR e-therapy* OR "text therap*" OR "internet-based therap*" OR teletherap* OR telecounsel*):ti,ab,kw (Word variations have been searched) 3534*

*#21 MeSH descriptor: [Distance Counseling] explode all trees 23*

*#22 MeSH descriptor: [Telemedicine] explode all trees 3329*

*#23 #20 OR #21 OR #22 6349*

*#24 #23 OR #19 1264128*

*#25 (smartphone OR application OR app OR mobile* OR iphone OR "smart phone"):ti,ab,kw (Word variations have been searched) 101886*

*#26 #25 OR #2 OR #3 102600*

*#27 #26 AND #8 AND #12 AND #15 AND #24 160*

Embase search strategy

*#21. ((('smartphone'/exp OR smartphone OR 198 20 Dec 2022 'application'/exp OR application OR app OR  mobile* OR 'iphone'/exp OR iphone OR 'smart phone'/exp OR 'smart phone') OR ('mobile phone'/exp OR 'mobile application'/exp)) AND ((mental OR depress* OR anxiety) OR ('mental health'/exp OR 'depression'/exp)) AND ((youth* OR 
'young adult*' OR teen*) OR ('adolescent'/exp OR 'young adult'/exp)) AND ((text* OR message* OR chat*) OR 'text messaging'/exp) AND (((therap* OR counsel* OR intervention*) OR ('psychotherapy'/exp OR 'counseling'/exp)) OR (('online counsel*' OR telehealth* OR 'e therapy*' OR 'text therap*' OR 'internet-based therap*' OR teletherap* OR telecounsel*) OR ('e-counseling'/exp OR 'telemedicine'/exp)))) AND (2017:py OR 2018:py OR 2019:py OR 2020:py OR 2021:py OR 2022:py)*
