# Supplementary material for: Phone-Based Text Therapy for Youth Mental Health: Rapid Review
Source: Interact J Med Res. 2023 Dec 14;12:e47250. doi: 10.2196/47250 (PMC10755647; doi:10.2196/47250)
Supplement: Multimedia Appendix 3 [file ijmr_v12i1e47250_app3.docx]

**Multimedia Appendix 3: Quality Assessments**

Table S1: Appraisal of Uncontrolled Studies using Quality Assessment Tool for Quantitative Studies from EPHPP (17)

|  | Criteria | | | | | | |
| --- | --- | --- | --- | --- | --- | --- | --- |
|  | Selection Bias | Study Design | Confounders | Blinding | Data Collection | Withdrawals and Drop-Outs | Global Rating for Paper |
| Jimenez et al (2022) | Moderate | Moderate | Moderate | Weak | Strong | Strong | Moderate |
| Goldin et al (2019) | Moderate | Moderate | Moderate | Weak | Strong | Moderate | Moderate |
| Chyzzy et al (2020) | Moderate | Strong | Moderate | Weak | Strong | Strong | Moderate |

Table S2: Quality Assessment of randomised control trials using Risk of Bias in Randomised trials (Rob-2) tool (16)

|  | Criteria | | | | |
| --- | --- | --- | --- | --- | --- |
| RCTs | Randomisation | Deviations from intended interventions | Missing outcome data | Measurements of outcome | Selection of reported results |
| Lindqvist et al (2020) | Low risk | Moderate risk  (no blinding) | Low risk | Moderate risk  (no inter-reliability measured) | Low risk |

Table S3: Quality Assessment of cross sectional studies using Cross-sectional studies: Appraisal Tool for Cross-Sectional Studies (AXIS) (19)

|  | Criteria | | | | | | | | | | | | | | | | | | | |
| --- | --- | --- | --- | --- | --- | --- | --- | --- | --- | --- | --- | --- | --- | --- | --- | --- | --- | --- | --- | --- |
| Question | 1 | 2 | 3 | 4 | 5 | 6 | 7 | 8 | 9 | 10 | 11 | 12 | 13 | 14 | 15 | 16 | 17 | 18 | 19 | 20 |
| Toscos et al (2018) | Y | Y | N | Y | Y | Y | N | Y | Y | Y | Y | Y | N | N | Y | Y | Y | Y | Y | Y |

Table S4: Quality Assessment of qualitative study using JBI Critical Appraisal Checklist for Qualitative Research (21)

|  | Criteria | | | | | | | | | |
| --- | --- | --- | --- | --- | --- | --- | --- | --- | --- | --- |
| Qualitative | Q1 | Q2 | Q3 | Q4 | Q5 | Q6 | Q7 | Q8 | Q9 | Q10 |
| Fai Yip et al (2021) | Y | Y | Y | Y | Y | N | N | Y | Y | Y |

Table S5: Quality Assessment of ecological studies using modification of STROBE from Dufault and Klar (22)

|  | Criteria | | | | |
| --- | --- | --- | --- | --- | --- |
| Ecological | Title and abstract | Objectives | Study Design | Participants | Limitations |
| Thompson et al (2018) | Y | Y | Y | Y | Y |
